# Emodepside has sex-dependent immobilizing effects on adult *Brugia malayi* due to a differentially spliced binding pocket in the RCK1 region of the SLO-1 K channel

**DOI:** 10.1371/journal.ppat.1008041

**Published:** 2019-09-25

**Authors:** Sudhanva S. Kashyap, Saurabh Verma, Denis Voronin, Sara Lustigman, Daniel Kulke, Alan P. Robertson, Richard J. Martin

**Affiliations:** 1 Department of Biomedical Sciences, Iowa State University, Ames, Iowa, United States of America; 2 Laboratory of Molecular Parasitology, Lindsley F. Kimball Research Institute, New York Blood Center, New York, New York, United States of America; 3 Bayer Animal Health GmbH, Drug Discovery and External Innovation, Leverkusen, Germany; McGill University, CANADA

## Abstract

Filariae are parasitic nematodes that are transmitted to their definitive host as third-stage larvae by arthropod vectors like mosquitoes. Filariae cause diseases including: lymphatic filariasis with distressing and disturbing symptoms like elephantiasis; and river blindness. Filarial diseases affect millions of people in 73 countries throughout the topics and sub-tropics. The drugs available for mass drug administration, (ivermectin, albendazole and diethylcarbamazine), are ineffective against adult filariae (macrofilariae) at the registered dosing regimen; this generates a real and urgent need to identify effective macrofilaricides. Emodepside, a veterinary anthelmintic registered for treatment of nematode infections in cats and dogs, is reported to have macrofilaricidal effects. Here, we explore the mode of action of emodepside using adult *Brugia malayi*, one of the species that causes lymphatic filariasis. Whole-parasite motility measurement with Worminator and patch-clamp of single muscle cells show that emodepside potently inhibits motility by activating voltage-gated potassium channels and that the male is more sensitive than the female. RNAi knock down suggests that emodepside targets SLO-1 K channels. We expressed *slo-1* isoforms, with alternatively spliced exons at the RCK1 (Regulator of Conductance of Potassium) domain, heterologously in *Xenopus laevis* oocytes. We discovered that the *slo-1f* isoform, found in muscles of males, is more sensitive to emodepside than the *slo-1a* isoform found in muscles of females; and selective RNAi of the *slo-1a* isoform in female worms increased emodepside potency. In *Onchocerca volvulus*, that causes river blindness, we found two isoforms in adult females with homology to *Bma-*SLO-1A and *Bma-*SLO-1F at the RCK1 domain. *In silico* modeling identified an emodepside binding pocket in the same RCK1 region of different species of filaria that is affected by these splice variations. Our observations show that emodepside has potent macrofilaricidal effects and alternative splicing in the RCK1 binding pocket affects potency. Therefore, the evaluation of potential sex-dependent effects of an anthelmintic compound is of importance to prevent any under-dosing of one or the other gender of nematodes once given to patients.

## Author summary

Emodepside is an anthelmintic drug that is used for treatment of nematode infections in cats and dogs. A potassium channel, SLO-1, has been implicated as a target for emodepside in the nematode model *Caenorhabditis elegans*. The anthelmintic effects of emodepside has been demonstrated against filariae, but its molecular mode of action requires further elucidation. Here, we show that emodepside activates SLO-1 channels in the human filaria, *Brugia malayi*, one of the causative agents of lymphatic filariasis. Emodepside is more potent on male than on the female *B*. *malayi*. Worms where *slo-1* was knocked down were less susceptible to emodepside. Alternative splicing of the *slo-1* gene in the region encoding the RCK1 domain, results in the expression of SLO-1F and SLO-1A in female worms and SLO-1F alone in male worms. SLO-1F expressed in *Xenopus laevis* oocytes was more sensitive to emodepside than SLO-1A or a combination of SLO-1A and SLO-1F. Selective knockdown of *slo-1a* in female worms increased their emodepside sensitivity, so their sensitivity resembled male worms. Emodepside binds to the RCK1 region of SLO-1 in *in-silico* protein interaction studies of the SLO-1 sequences of different filariod species. This is the first report demonstrating a gender-specific effect of emodepside correlating directly to the expression of alternatively spliced isoforms of SLO-1. This study emphasizes that the gender-dependent effects should always be considered when evaluating the efficacy profile of an anthelmintic drug candidate.

## Introduction

### Emodepside as a macrofilaricide

Filariae are parasitic nematodes that reside in the definitive host which are always mammals. The adult filariae are referred to as macrofilariae. The adult females release microscopic juveniles, microfilariae, which are then ingested during a blood meal by the arthropod vectors. These microfilariae develop in the arthropod host to the infectious third-stage larvae which can then be transmitted to the mammalian host during a subsequent blood meal. The filaroide species that are responsible for most of the morbidity are *Wuchereria bancrofti*, *Brugia malayi* and *Brugia timori* that cause lymphatic filariasis (including elephantiasis), and *Onchocerca volvulus* that causes river blindness (onchocerciasis) [1]. These diseases occur mostly in sub-Saharan Africa and Southeast Asia and affect 168 million people. Some 120 million people are infected with lymphatic filariases and around 20 million people are affected by onchocerciasis. The diseases are not usually fatal, but they produce distressing symptoms including, swollen limbs, blindness, reduced work productivity, social rejection and suppression of immune responses to diseases like, malaria and tuberculosis. For over 25 years now, the strategy for controlling filarial diseases has relied on Mass Drug Administration (MDA) programs directed by the World Health Organization using donated drugs to reduce the transmission and morbidity of these filarial diseases. The MDA programs have been successful in several countries, but the filarial diseases still persist, and have not been eliminated [2].

The existing drugs that are now available for MDA (albendazole and ivermectin or diethylcarbamazine for lymphatic filariasis, and ivermectin alone for onchocerciasis are effective against the early larval stages of the parasites (microfilariae), but they do not kill the adults (macrofilariae) efficiently. These repeated annual treatments in MDA programs were modelled to take more than 6–8 years to control lymphatic filariasis, and >15 years to eliminate onchocerciasis because the adults persist and continue to release microfilaria between drug treatments and to spread infection. To accelerate the control measures, there is a real need to identify effective macrofilaricides. The Drugs for Neglected Diseases initiative (DNDi) development program has focused on macrofilaricide drug candidates from repurposing libraries and have included drugs that have been registered for animal health [3]. Emodepside is a veterinary drug that has effects on adult filarial nematodes [4] and has completed first-in-human safety and tolerability studies in healthy volunteers with the purpose of being developed for the treatment of onchocerciasis [5]. Emodepside is a semisynthetic derivative of the cyclooctadepsipeptide PF1022A that was isolated from *Rosellinia spp*., a fungus that grows on *Camellia japonica* [6]. Emodepside is effective for treating animal nematode parasites that are resistant to other common anthelmintics [7]. It inhibits motility in nematodes, via an effect that may be mediated through GABA_A_ receptors [8, 9], lathrophilin receptors [10, 11] or SLO-1 K channels [12–15]. The general consensus is that SLO-1 is the main target of emodepside in nematodes [16]. Detailed modes of action studies in filarial nematodes are required to advance the evaluation of emodepside as a macrofilaricide.

The effects of emodepside against filariae are dose- and species-dependent. In studies using *Litomosoides carinii* (syn. *L*. *sigmodontis*), *Acanthocheinlonema viteae*, *Brugia malayi*, *Brugia pahangi*, *Onchocerca gutturosa* and *Onchocerca lienalis*, the adults of *Brugia* spp. emerged as the dose-limiting stage where emodepside was least potent [17, 18]. Here we study the mode of action of, and sensitivity to, emodepside using the dose-limiting filarial nematode, *B*. *malayi*. RNAi knock down experiments demonstrated that emodepside targets the SLO-1 K channels. We expressed *slo-1* isoforms with alternatively spliced exons in the RCK1 domain heterologously in *Xenopus laevis* oocytes and observed that the *slo-1f* splice variant, found in muscles of males, is more sensitive to emodepside than the *slo-1a* splice variant found in muscle of females. We also demonstrate selectively knocking down the *slo-1a* splice variant in female *B*. *malayi* increases emodepside efficacy, signifying a dominant suppressor role for SLO-1A. Further, we show that similar alternatively spliced isoforms are expressed in *O*. *volvulus*.

*In silico* modeling identified a putative emodepside binding pocket in the same RCK1 region of different species of filariae that is affected by these splice variations. Our observations show the potent macrofilaricidal effects of emodepside are influenced by sex-linked splice variants in the RCK1 binding pocket of SLO-1. The sex of the filarial target is therefore an important therapeutic variable to consider when evaluating a dose-regimen for treatments during clinical trials [5].

## Results

### Emodepside has potent gender-dependent effects on adult *Brugia malayi*

**Fig 1:**
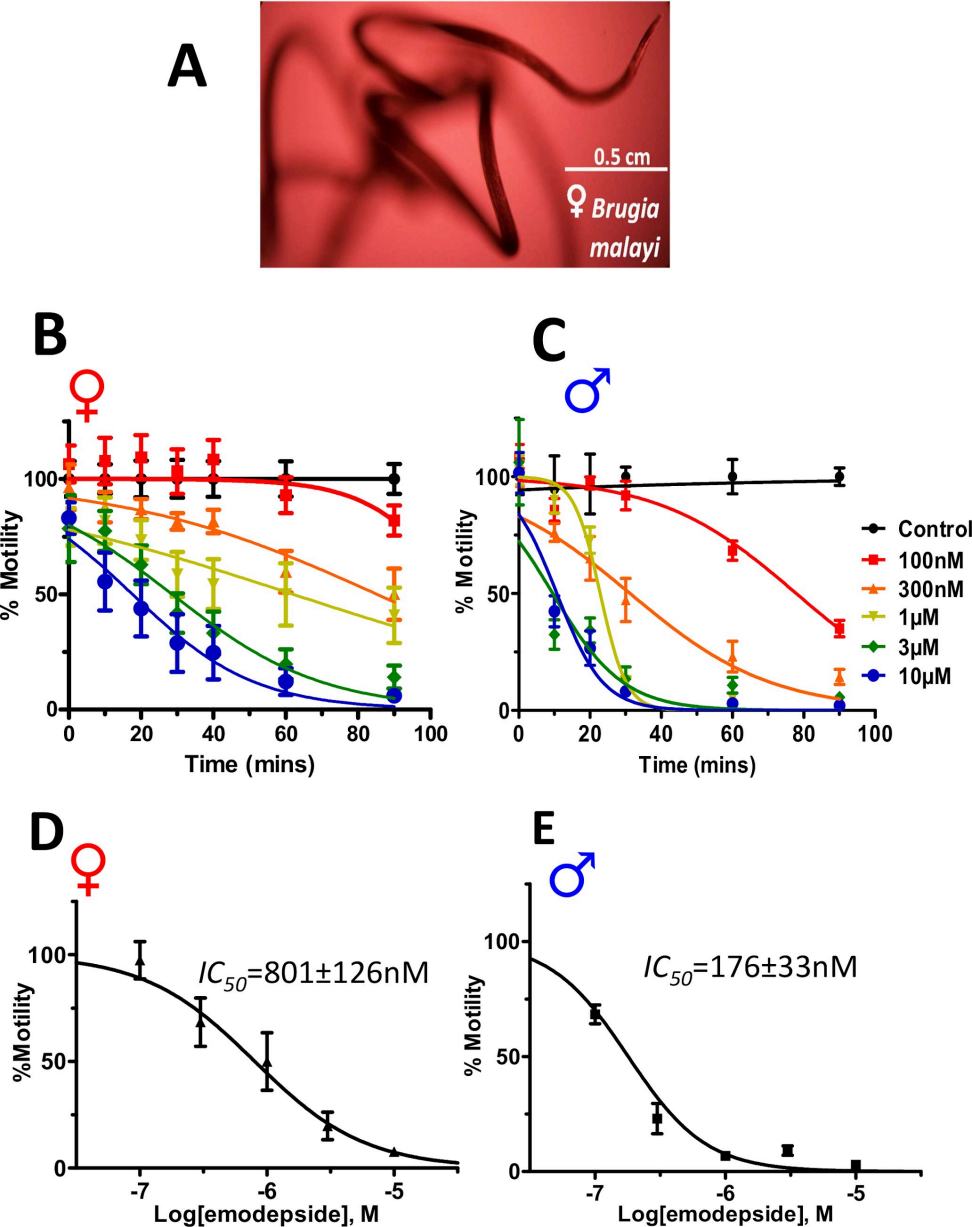
Emodepside causes inhibition of motility of whole adult *Brugia malayi* that is concentration-, time-, and sex-dependent. **A:** Representative micrograph of an untreated adult female *B*. *malayi*. The worm is characteristically coiled and was showing continuous vigorous vibrating movements. **B:** (**female symbol**) Concentration- and time-dependent emodepside inhibition of motility on adult *B*. *malayi* females and **C: (male symbol**) males. Motility plotted at 60 minutes for both females and males (**D: female symbol:** *IC*_*50 (female*),_ 801 ± 126nM (SEM)) and males (**E: male symbol:** *IC*_*50 (male)*,_ 176 ± 33 nM (SEM)). Each treatment group was normalized to the motility at time 0. N = 12, over two biological replicate experiments. The female *IC*_*50*_ was significantly higher than the male (p<0.001; 2-way ANOVA). The male is more sensitive to emodepside than the female.

We selected *B*. *malayi*, Fig 1A, a ‘dose-limiting species’, to pursue the investigation of the effects of emodepside. Initially, we used motility assays to determine emodepside *IC*_*50*_ values from concentration-response relationships of adult males and females. Emodepside causes flaccid paralysis of the adult worm. Both the male and female worms show reduced motility as early as 10 minutes exposure at the highest concentration tested, 10μM. Fig 1B & 1C shows that the immobilizing effects of emodepside were concentration-, time- and sex-dependent. When we plotted the emodepside- concentration motility-response at 60 minutes, the male is 4.5x more sensitive to emodepside than the female, Fig 1D & 1E: the male *IC*_*50*_ was 176±33 nM and the female *IC*_*50*_ was 801±126 nM (p<0.001; 2-way ANOVA; n = 12).

The gender-difference in sensitivity is therapeutically relevant if the female worm remains after treatment to release microfilariae allowing continued transmission of the infection. One possible explanation for the gender-linked sensitivity was the bigger size of the female worms (length 43 to 55 mm) compared to the males worms (length 13 to 23 mm), limiting the bioavailability of emodepside within the female worm. To investigate the direct effect of emodepside on muscle cells we used dissected preparations [19, 20] from female and male worms and recorded the responses in exposed muscle cells using patch-clamp.

### Emodepside increases outward voltage-activated & holding currents and is more potent on male muscle cells

When we used a voltage-step protocol to activate voltage-sensitive potassium currents in muscle cells, we found that these currents increased in the presence of emodepside. S1A & S1B Fig shows representative currents activated by depolarizing steps from a holding potential of -40mV in female worms. The major effect of emodepside on the potassium current activation curve was to increase *G*_*max*_ from 14±2 pS to 23±1 pS (p<0.001; Student’s *t*-test; n = 5), suggesting an increase in the number of channels opening and/or the open probability of the channels. There was only a small increase in the voltage sensitivity with the *V*_*50*_ changing from 8±1 mV to -2±1 mV (p<0.001; n = 5; paired *t-test*; S1C Fig), showing that a change in sensitivity of the channels to depolarization is not a major mechanism of action of emodepside.

**Fig 2:**
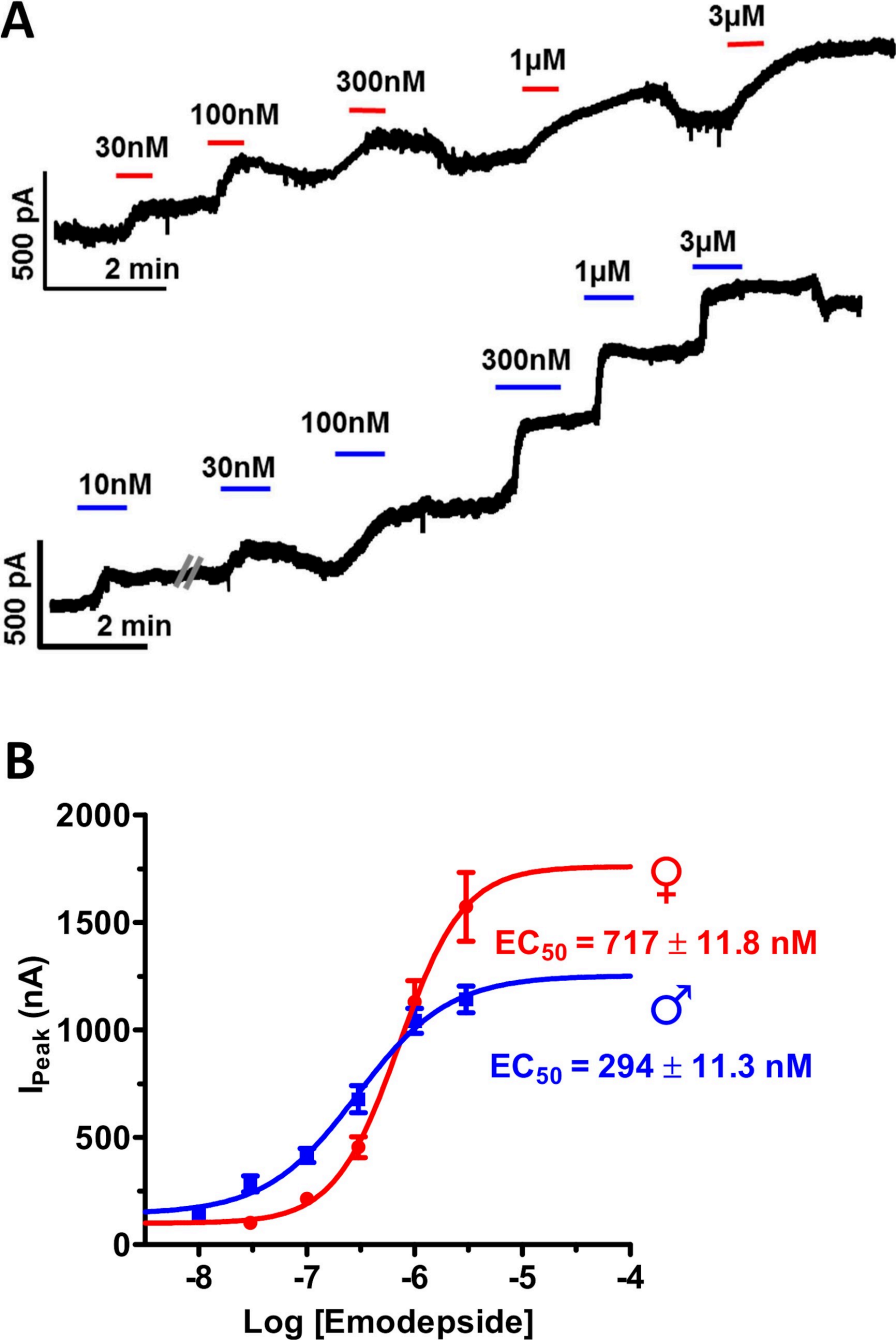
Emodepside produces concentration-dependent outward currents in female and male *B*. *malayi* muscle cells. **A**: Representative current traces of the concentration-dependent emodepside activated standing outward currents from muscle cells of female *B*. *malayi*. Cells were whole-cell patch-clamped at -40mV and each concentration of drug was applied consecutively in increasing concentrations (30nM to 3μM) and were allowed to plateau before washing and the next application. **B:** Emodepside activated outward currents from muscle cells of male *B*. *malayi*. **C**: Concentration-dependent mean current curves for emodepside activated outward currents. Red female symbol: *EC*_*50 (Female)*_ = 720 ± 12nM (n = 5), blue male symbol: *EC*_*50 (Male*)_ = 294 ± 11nM (n = 5). The male is more sensitive to emodepside than the female.

To compare the effects of emodepside on male and female muscle cells, we measured the increases in the outward standing currents at a potential near the resting membrane potential, -40mV, Fig 2A & 2B. The males were more sensitive to emodepside than the females. The *IC*_*50*_ of male muscle cells was significantly less (~2.5x) than that of female muscle cells: 294±11nM in males (n = 5) and 717±12nM (n = 5) in females (p<0.001; 2-way ANOVA). It is pointed out however that the *I*_*max*_s of female muscle cells were bigger than that of male muscle cells, which may be explained by the larger size of the female muscle and the activation of more SLO-1 channels. These *IC*_*50*_s and the differences between males and females are similar to the *IC*_*50*_s seen for the whole worm motility experiments (Fig 1C & 1D). The patch-clamp experiments showed that: a) the size of the worm and the cuticle barrier had little effect on emodepside potency; b) emodepside is more potent on male worm muscle cells than on female worm muscle cells; and c) emodepside affects *B*. *malayi* muscle by activating standing outward (hyperpolarizing) currents.

### Iberiotoxin, a selective SLO-1 K channel antagonist, inhibits emodepside currents

The parasite genome has several potassium channels and it is likely that more than one potassium channel type is expressed in somatic muscles. We used a selective SLO-1 K channel inhibitor, iberiotoxin [21], a toxin from the scorpion *Buthus tamulus* that binds selectively to the external surface of SLO-1 K channels. We found that iberiotoxin by itself had little effect on the standing current, S2A Fig, but significantly (p<0.005; Student’s paired *t-*test; n = 7) inhibited the emodepside-activated outward current, S2A & S2B Fig. This inhibition supports the hypothesis that the mode of action of emodepside in filariae is to activate SLO-1 K channels.

### Knock down of *slo-1* in adult females produces an emodepside resistant phenotype

**Fig 3:**
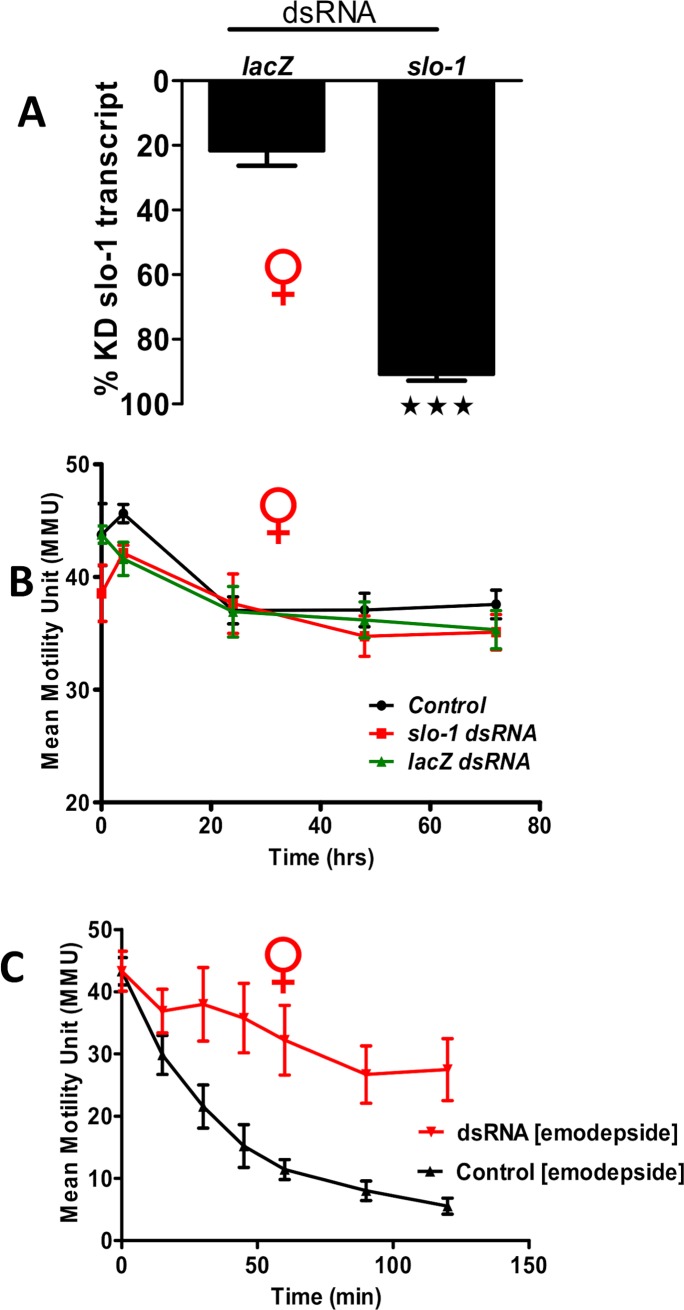
*slo-1* RNAi treated female worms are resistant to emodepside. **A:** Knock down of *slo-1* transcript after 72 hours of incubation was assayed using qPCR. Knock down of the *slo-1* transcript was 91 ± 2% and 22 ± 12% in worms treated with control *LacZ* dsRNA. (p<0.005, *t-*test, n = 6). **B:** Little or no change in Mean Motility Units (MMU) was observed in adult female, Red female symbol, *B*. *malayi* control worms, *lacZ*- and *slo-1*-dsRNA treated worms over 72 hours. **C:** Worms soaked in *slo-1* dsRNA show an emodepside resistance phenotype. The motility of the control worms was significantly inhibited (only 5% motility was observed after treatment with 300nM emodepside) after 120 minutes while the dsRNA treated worms were resistant to emodepside (2-way ANOVA: p<0.001; n = 24 over 4 biological replicates).

We knocked down the *slo-1* transcript in adult female *B*. *malayi* to see if we could produce a SLO-1 loss of function phenotype that shows resistance to emodepside. We used *slo-1* targeting dsRNA and were able to knock down its expression by 91±2% after 3 days (Fig 3A), which was significant (p<0.005; Student’s *t-*test; n = 6) when compared to the control worms treated with *lacZ* dsRNA that showed a 22±12% decrease in the *slo-1* transcript. The dsRNA treatments produced little change in motility over a period of 72 hours (Fig 3B). When we treated the *slo-1* knock down worms with 300nM emodepside and measured their motility over a period of two hours, we found that worms treated with *slo-1* specific dsRNA were resistant (two-way ANOVA; p<0.001) to emodepside as compared to *lacZ* dsRNA treated control worms (Fig 3C). These results show that emodepside requires SLO-1 K channels to paralyze adult *B*. *malayi*.

### *slo-1* splice variants expressed in *B*. *malayi* muscle cells

SLO-1 channels in filaria are highly conserved, but alternative splicing can produce channel isoforms [22] which could alter their pharmacology. In the *B*. *malayi* genome (parasite.wormbase.org) there are 5 predicted *slo-1* isoforms, a, b, c e and f, S3A Fig. Splice variants a and f are full length, b and c lack the first exon, while e is a truncated version. Between a, b, c and f, the splice sites are predicted to be in exon 2 and in exon 14 of the *slo-1* gene. To identify the splice variants that are present in *B*. *malayi*, we made the primers BSloR1 and BSloR2 to cover and amplify this region from *B*. *malayi* cDNA. We amplified the 5’ end of the *slo-1* gene using 5’ splice leader 2 primer and were able to amplify a single band confirming the expression of just two splice variants, *slo*-1a & *slo*-1f (S3B and S3C Fig).

We have shown the greater potency of emodepside on male worms compared to female *B*. *malayi* worms (Figs 1 and 2). We hypothesized that this difference may arise from differences in the expression of *slo-1* splice variants between the sexes. To test this, we used a NcoI restriction site to cut *slo-1a* specifically at exon 14; we amplified full-length *slo-1* transcript from cDNA collected via the patch-pipettes from single muscle cells of both male and female adult worms and cut both the amplicons using the restriction enzyme NcoI. We found clear differences in the expression of the *slo-*1 splice variants between the sexes of adult *B*. *malayi* (S3C Fig): splice variants *slo-1a* and *slo-1f* were both expressed in female worms, but only *slo-1f* was expressed in male worms.

### SLO-1F is more sensitive than SLO-1A to emodepside

**Fig 4:**
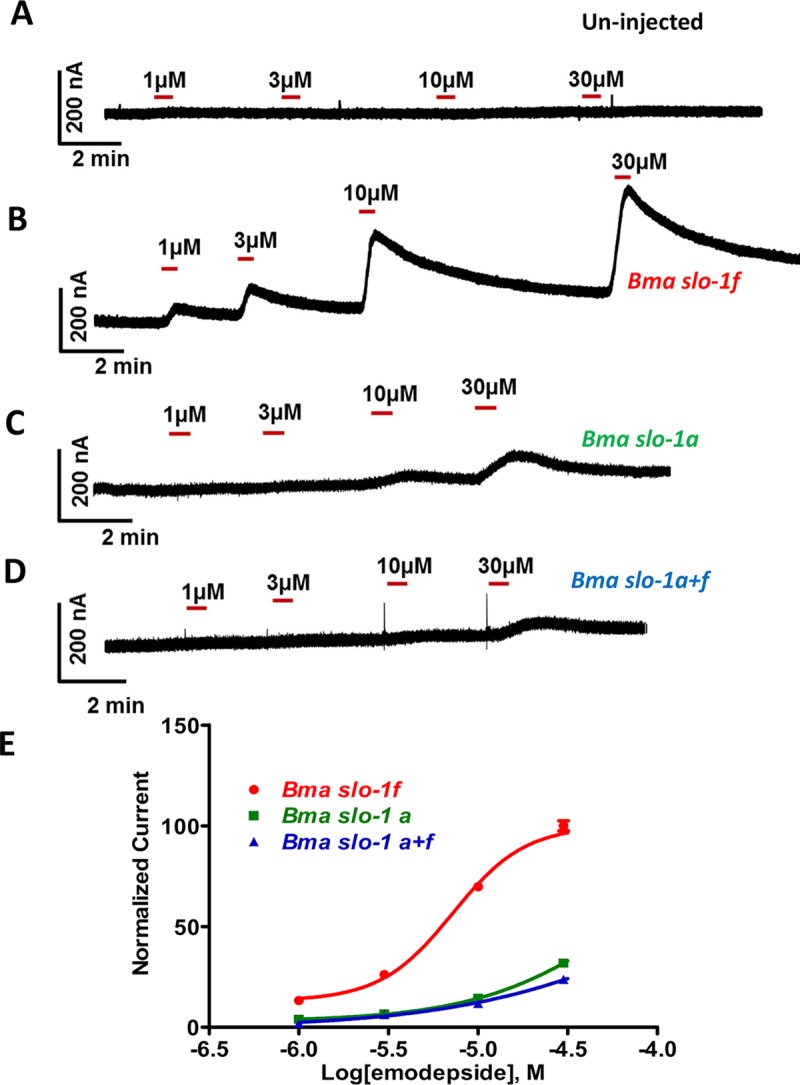
Emodepside induced outward currents in *Xenopus laevis* oocyte expressed *slo-1* channels. Representative traces for two-electrode voltage clamp (TEVC) experiments on *X*. *laevis* oocytes injected with different isoforms of *slo-1* cRNA and clamped at +20mV. **A:** Lack of effects of emodepside on naïve un-injected oocytes. **B:** Effects of emodepside on *B*. *malayi slo-1f* cRNA (15ng) injected oocytes. **C:** Effects of emodepside on *slo-1a* cRNA (15ng) injected oocytes. **D:** Effects of emodepside on *slo-1a + slo-1f* cRNA (7.5ng each) injected oocytes. **E:** Concentration-dependent curves for emodepside induced currents from *X*. *laevis* oocytes injected with *slo-1f* (*EC*_*50*_ *= 5* ± 1 μM; n = 7), *slo-1a* and *slo-1a+f* cRNA (*EC*_*50*_>30μM; n = 7 for both).

We cloned both *Bma*-*slo-1a* and *Bma*-*slo-1f* into an expression vector (pTB207) to express the channels in *Xenopus laevis* oocytes. We recorded evoked currents in response to emodepside using two-electrode voltage-clamp with oocytes held at +20mV. Control experiments on water-injected oocytes produced no response (Fig 4A). Robust concentration-dependent current responses that had peak currents of over 400nA were seen with oocytes injected with *slo-1f* (Fig 4B). When we expressed *slo-1a*, or *slo-1a* with *slo-1f* (Fig 4C & 4D), we found much smaller, but still detectable currents. The *EC*_*50*_ for SLO-1F was 5±1μM (n = 7), which was significantly (p<0.001; Student’s *t-*test) lower than that of the SLO-1A and the SLO-1F + SLO-1A combination (*EC*_*50*_ > 30μM; n = 7) for both, Fig 4E. These observations show that emodepside displays different potencies on different splice variants of *Bma-slo-1*. We point out that the emodepside *EC*_*50*_*s* from the oocyte experiments are higher than those obtained from the whole worm motility and muscle patch-clamp experiments, which might be explained by the absence of interacting proteins like ISLO-1 (Interactor with SLO-1), BKIP-1 (BK channel interacting protein) [16, 23].

### Knockdown of *slo-1a* increases emodepside potency in adult female *B*. *malayi*

**Fig 5:**
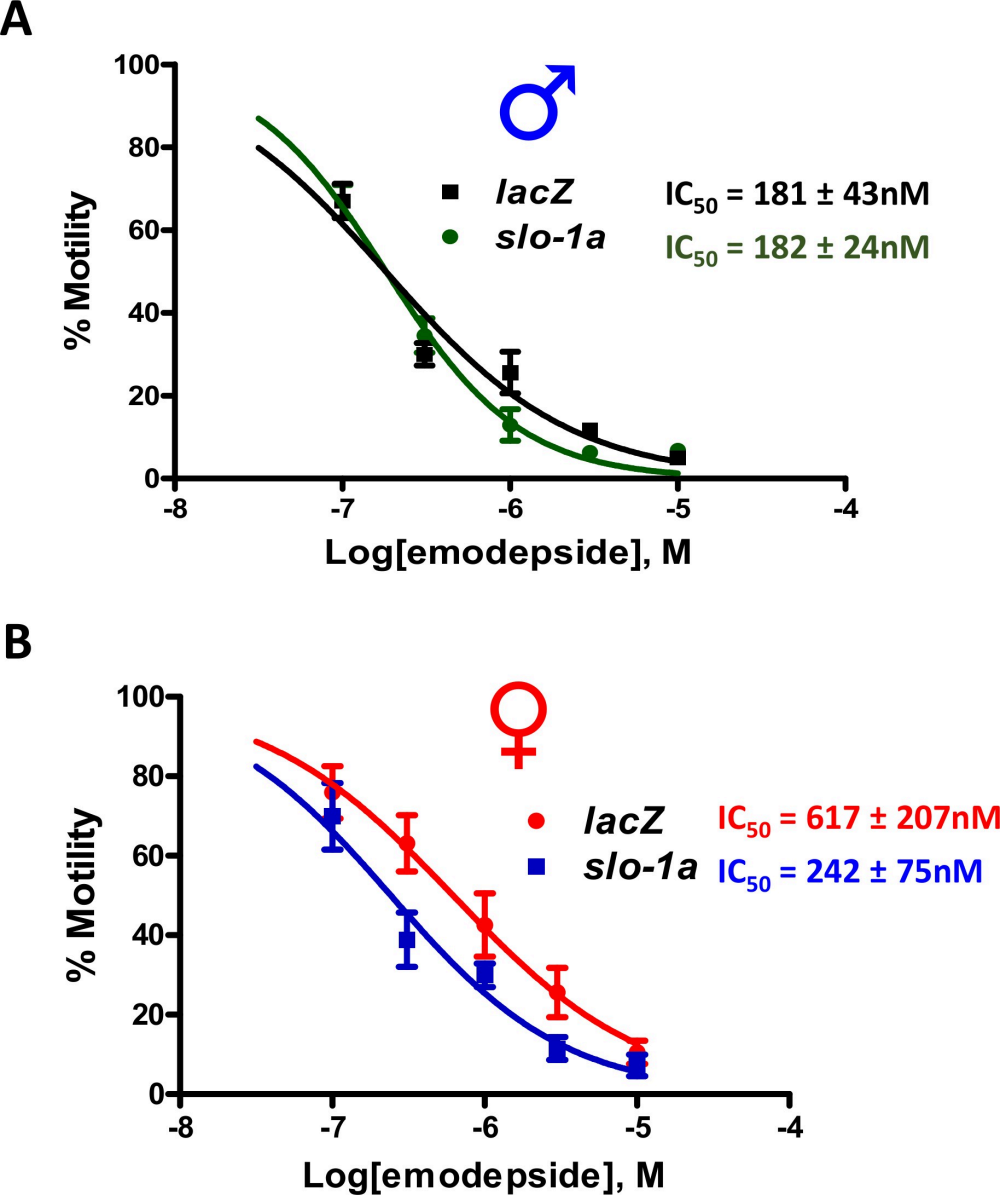
RNAi of *slo-1a* increases the potency of emodepside. **A:** Effects of RNAi knock down of *slo-1a* in adult male. Off target lacZ dsRNA was used as control. *IC*_*50*_*s*: lacZ Blue male symbol: 181±43nM; *slo-1a* Blue male symbol = 182±24nM. **B:** Effects of *slo-1a* knock-down on adult female. *IC*_*50*_*s*: *lacZ* Red female symbol: 617±207nM; *slo-1a* Red female symbol: 242±75nM. *IC*_*50*_s for both *slo-1a* (Red female symbol) and (Orange male symbol) were significantly lower than the *IC*_*50*_ of the control *lacZ* Red female symbol. (2-way ANOVA; p<0.05; N = 8 over two biological replicates). Thus, reduced expression of *slo-1a* splice variants in females increases the potency of emodepside.

We demonstrated earlier that knock down of *slo-1* causes a resistance to emodepside phenotype (Fig 3C) due to the loss of both the *slo-1a* and *slo-1f* splice variants. As shown in S3A Fig, the difference in the homology between SLO-1A and SLO-1F is in a single alternatively spliced exon. Of the 37 amino acids in the region of exon-14, 11 amino acids are different because of the splice variation. To further test the hypothesis that expression of *slo-1a* splice variant reduces the emodepside potency, the region of exon-14 of *slo-1a* was synthesized as single strands, annealed and dsRNA was synthesized. We then selectively knocked down *slo-1a* in female *B*. *malayi*. Female worms treated with off-target *lacZ* and male worms treated with *slo-1a* specific dsRNA were used as controls. Worms were soaked in the dsRNA for 72 hours and were then treated with increasing doses of emodepside. Emodepside was then more potent (*IC*_*50*_: 242 ± 75nM) in female worms treated with *slo-1a* dsRNA (Fig 5A & 5B): the *IC*_*50*_ was 3 times lower than in control female worms (*IC*_*50*_: 617 ± 207nM) treated with *lacZ* dsRNA (2-way ANOVA; p<0.05; n = 8). We point out that male worms treated with *slo-1a* dsRNA showed little change in their *IC*_*50*_ (181 ± 43nM, Fig 1E vs 182 ± 42nM, Fig 5A & 5B). We obtained cDNA from both male and female worms and used restriction enzymes *PvuII* to selectively cut *slo-1f*, and *NcoI* to selectively cut *slo-1a*. We performed qPCR on the cleaved DNA and confirmed greater than 80% knockdown (P<0.01, Student’s *t-*test, n = 5) of *slo-1a* transcript in female worms (S4A Fig) but no significant change of the *slo-1f* in the female or male worms (S4A & S4B Fig). We point out that *PvuII* and *NcoI* does not cleave the region of *gapdh* we amplified for quantification.

### Putative emodepside binding pocket on RCK1

**Fig 6:**
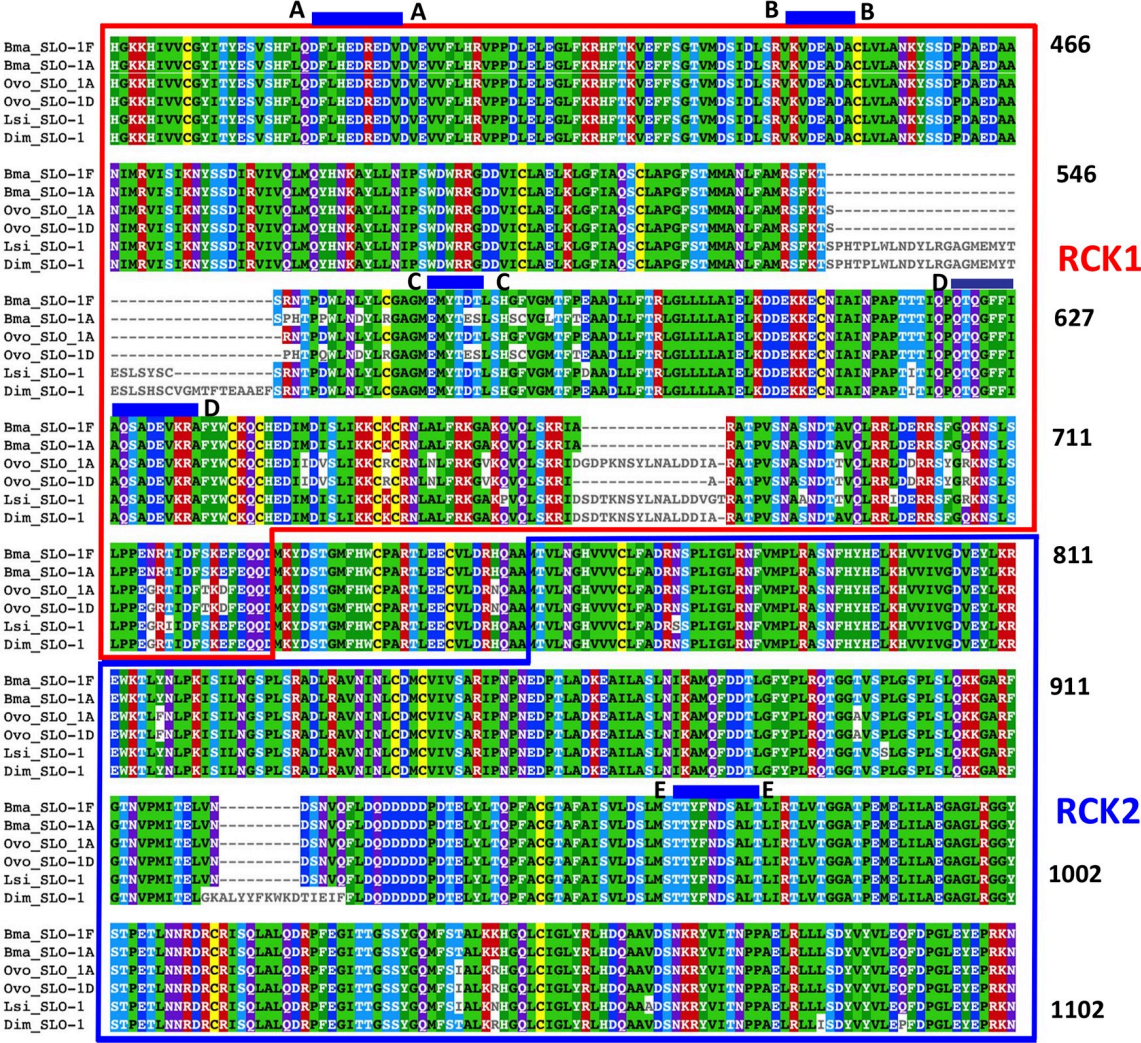
Emodepside binding loops on the cytoplasmic domains of SLO-1. Multiple sequence alignment of the cytoplasmic domains of *B*. *malayi* (slice variants, F & A), *O*. *volvulus* SLO-1 (splice variants A & D), *L*. *sigmodontis*, and *D*. *immitis*. The amino acids are numbered from the beginning of the RCK1 region (HGK) and show the emodepside binding loops: A (389–400), B (442–449), C (562–567), D (622–638) on the RCK1 region and E (965–973) on the RCK2 region.

**Fig 7:**
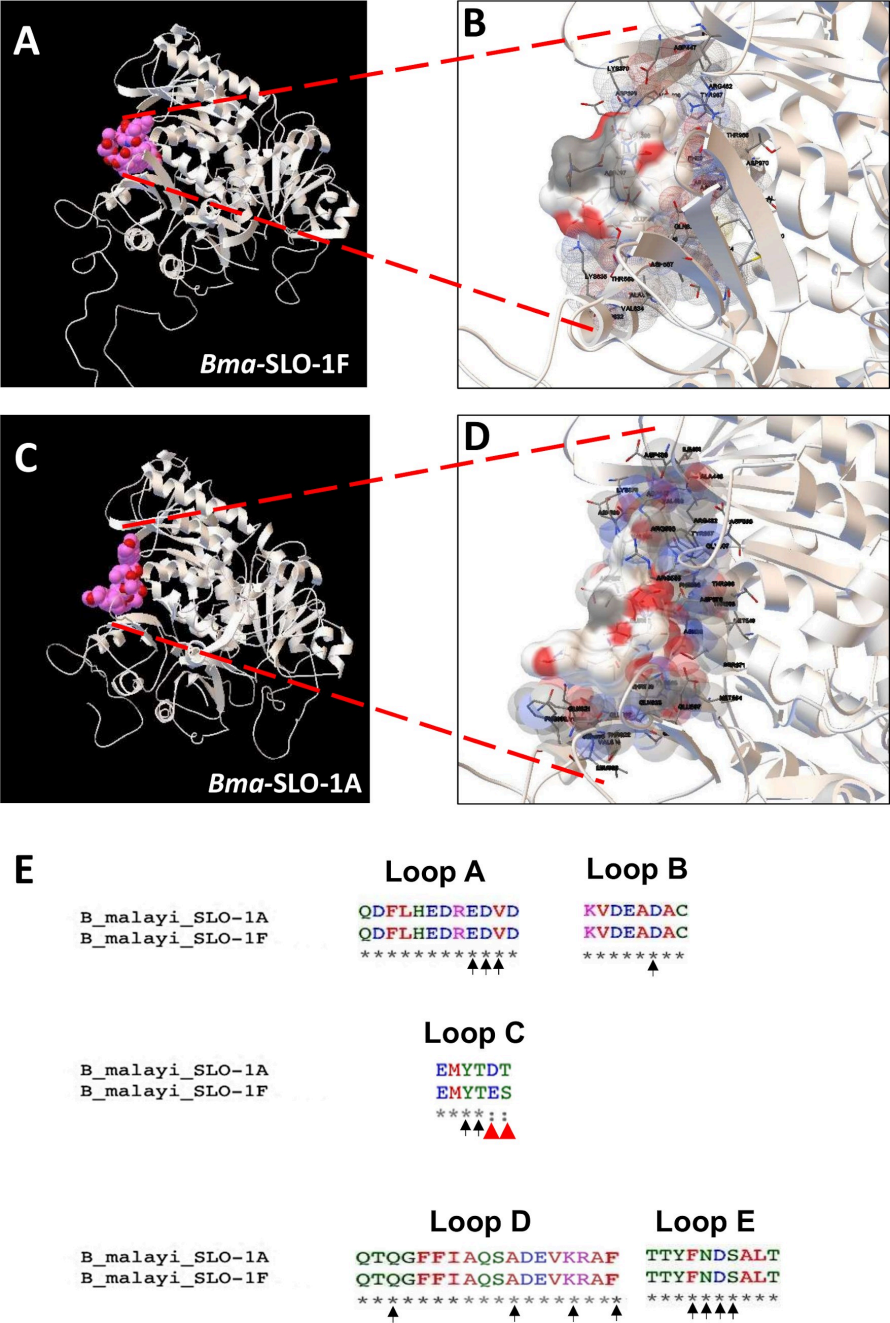
*In-silico* analysis of emodepside binding to the cytoplasmic domains of SLO-1 of filaria. Homology models of the cytoplasmic domains of filarial SLO-1s with docked best fitting conformers of emodepside; for clarity only the single subunit cytoplasmic region is shown. **A:** & **B:** *B*. *malayi* SLO-1F; **C:** & **D:** *B*. *malayi* SLO-1A. Shows a detailed view of the putative binding pocket of *B*. *malayi* SLO-1F and SLO-1A with emodepside docked. **E:** Shows amino acid sequences (in arrows) of different loops where emodepside physically interacts or is in close contact of SLO-1 in *B*. *malayi*. The red arrows indicate amino acids that interact with emodepside and are different in SLO-1A and SLO-1F.

We have shown that the potency of emodepside differs with the splice variants of *slo-1* in *B*. *malayi*. The divergence in the sequence between SLO-1A and SLO-1F is found in the cytoplasmic RCK1 domain, suggesting that this region is involved in emodepside binding. We performed *in-silico* binding experiments and tested the docking of the three most thermodynamically stable structures of emodepside to modeled structures of the cytoplasmic domain of SLO-1F and SLO-1A isoforms of *B*. *malayi*. We found (Figs 6 & 7A–7E) that emodepside binds most favorably within the same pocket of *Bma*-SLO-1, which was formed by five putative binding loops (loop A: amino acids 389–400 defined using *Bma-*SLO-1F; loop B: 442–449; loop C: 562–567; loop D: 622–638; and loop E: 965–973, Figs 6 and 7E and Table 1). Loops A-D are present in the RCK1 region. The amino acid differences in loop C (see E566D and S567T; red arrows, Fig 7E) due to alternative splicing in exon 14 changed the binding energy of SLO-1F from -6.23 kCal/mol to -4.45 kCal/mol of SLO-1A. Loop E is present in the RCK2 region near the calcium bowl (amino acids 930–938) region. Fig 7E shows with vertical arrows amino acids on these loops that directly interacts with emodepside.

**Table 1:** Amino acids that interact with emodepside in bold are residues in C-loop that are different due to alternative splicing. The table shows the amino acids recognized by PYMOL in different filariae that are close and physically interacts with emodepside. Highlighted in red are residues in C-loop that are different due to alternative splicing. *Brugia*: *Bma* SLO-1A & *Bma* SLO-1F; for *Onchocerca volvulus*: *Ovo* SLO-1A & Ovo SLO-1D; for equivalent sequences of *Dirofilaria immitis*: Dim SLO-1; and for *Litomosoides sigmodontis*: *Lsi* SLO-1.

*Bma* SLO-1A	*Bma* SLO-1F	*Ovo* SLO-1A	Ovo SLO-1D	Dim SLO-1	Lsi SLO-1
GLU396	GLU396	GLU396	GLU396	GLU66	GLU155
ASP397	ASP397	ASP397	ASP397	ASP67	ASP156
VAL398	VAL398	VAL398	VAL398	VAL68	VAL157
ASP447	ASP447	ASP447	ASP447	ASP117	ASP206
TYR565	TYR565	TYR565	TYR565	TYR274	TYR352
THR566	THR566	THR566	THR566	THR275	THR353
**ASP567**	**GLU567**	**GLU567**	**ASP567**	**ASP276**	**ASP354**
**THR568**	**SER568**	**SER568**	**THR568**	**THR277**	**THR353**
GLN623	GLN623	GLN623	GLN623	GLN332	GLN410
ALA631	ALA631	ALA631	ALA631	ALA340	ALA418
LYS635	LYS635	LYS635	LYS635	LYS344	LYS442
PHE638	PHE638	PHE638	PHE638	PHE347	PHE445
PHE968	PHE968	PHE983	PHE968	PHE701	PHE771
ASN969	ASN969	ASN984	ASN969	ASN702	ASN772
ASP970	ASP970	ASP985	ASP970	ASP703	ASP773
SER971	SER971	SER986	SER971	SER704	SER774

We investigated the SLO-1 RCK1 region of other filaria species to explore the binding of emodepside. In *O*. *volvulus*, the filaria that causes river blindness, we made similar observations. *O*. *volvulus* has 5 predicted *slo-*1 splice variants (Wormbase, OVOC4127a –d & f; e is a truncated form with the RCK domains absent). When the cDNA from female *O*. *volvulus* was amplified between exons 9 and 19 of *slo-1* only two splice variants were expressed: *Ovo*-SLO-1A and *Ovo*-SLO-1D (S5 Fig). *In silico* experiments revealed that emodepside binds most favorably to the same regions, loops A-E in *O*. *volvulus* as in *B*. *malayi* (S6 Fig). The emodepside binding energies being -5.08 kCal/mol to Ovo-SLO-1A and -5.60 kCal/mol to Ovo-SLO-1D. Interestingly, both of these energies are higher than the emodepside binding energy for the limiting SLO-1A splice variant of *B*. *malayi* and imply a greater potency of emodepside in *O*. *volvulus*.

We also modelled the *in-silico* binding of emodepside to the SLO-1 Ks of the filaria, *Dirofilaria immitis* and *Litomosoides sigmodontis* using the available Parasite Wormbase (parasite.wormbase.org) sequences, Fig 6, although it lacks information about splice variants. Again, we found that emodepside binds most favorably to the same putative pockets defined by the same A-E loops (Figs 6 & S6).

## Discussion

### Emodepside as a macrofilaricide

DNDi in collaboration with Bayer AG have pursued the development of emodepside, originally a veterinary anthelmintic, with the aim of it being the first registered oral macrofilaricide treatment for a filarial disease, onchocerciasis, with DNDi planning to run a Phase II “proof-of-concept” clinical trial in sub-Saharan Africa investigating the safety and efficacy of the drug. It has been shown to be effective, albeit with varying potency, against other filarial nematode species in *in vitro* and *in vivo* models using rodents [4, 17, 18]. Here we show that emodepside has concentration-dependent activity even against the dose-limiting filarial species, *Brugia malayi*. An oral dose of 1.9mg/Kg emodepside produces a maximum plasma concentration of 150μg/L or 125nM in dogs with emodepside being distributed to all organs and the highest concentrations being found in the fat [24]. The *IC*_*50*_ for the adult cattle filaria, *O*. *gutturosa in vitro*, is estimated to be <1nM [4]. If 125nM concentrations of emodepside are achieved in humans following treatment, and *O*. *volvulus* is as sensitive to emodepside as *O*. *gutturosa*, emodepside is anticipated to be effective against onchocerciasis. The effectiveness and safety profile of emodepside against *B*. *malayi* has yet to be determined in humans. Our results predict that higher plasma concentrations of emodepside would be required against *B*. *malayi*.

### Splice-dependent effects and a putative binding site in the RCK1 region

We found that emodepside was more potent on *Bma-*SLO-1F than *Bma-*SLO-1A. We observe that there is alternative splicing in the RCK1 region in *B*. *malayi* (*slo-1a* and *slo-1f*) and that there are differences in the RCK1 amino acid sequences between the other filarial species as well. In the full-length SLO-1 splice variants of *B*. *malayi* and *O*. *volvulus*, we find the same alternatively spliced regions between exons 13–20 which encode the RCK1 region. We do not comment more specifically on alternatively spliced *slo-1* transcripts of *L*. *sigmodontis* due to the lack of sequence information in the database. Although there is a report of splice variants being expressed in *D*. *immitis* with alternatively spliced exons in the RCK1 region, very similar to *B*. *malayi* [22], we do not have the full-length sequences from the Wormbase Parasite database for *D*. *immitis* to use in our study. Nonetheless, we still find less homology between different filarial species in the RCK1 region compared to RCK2 (Fig 6). Given these differences in the RCK 1 regions, the reduced potency of emodepside in *B*. *malayi* and sensitivity of *Onchocerca* species, we looked *in-silico* at the RCK1 region for emodepside binding sites (Fig 6). We found that in all species of filaria examined that emodepside binds most favorably in the same pocket bound by five separate loops, A-E. Loops A-D are present in the RCK1 region (S6 Fig). Loop E is present in the RCK2 region adjacent to the Calcium Bowl. The *in* silico observations showing emodepside binding to the RCK1 region of *O*. *volvulus* further advances our knowledge of its effectiveness as a macrofilaricide for the treatment of river blindness.

Observations on a number of *C*. *elegans* SLO-1 mutants have also noted that amino-acid changes in the RCK1 region reduces emodepside potency, supporting the presence of an emodepside binding pocket in this region [15, 25]. Our observations do not rule out additional binding of emodepside to transmembrane regions of the channel in the lipid phase of the membrane; but we observed only a small change in the voltage-sensitivity of the channel that would, if large, indicate an effect mediated via the voltage-sensing domain in the membrane [26, 27].

### Reporting gender specific effects in parasites

We have observed that emodepside is more potent on male *B*. *malayi* than females and therefore the gender of nematodes has an effect as a variable on the outcome of emodepside treatment. Insufficient concentrations of emodepside would leave the female worms surviving treatment to continue their life cycle and the diseases. The National Institutes of Health (NIH) expects that scientists test for the possible role of gender as a biological variable in vertebrate animals and humans (Notice Number NOT-OD-15-102) but does not require reporting of gender as a variable on invertebrates, here parasitic nematodes. This study shows that gender is a significant factor for the effect of emodepside. Significant gender-dependent differences in the metabolism of anthelmintics has been observed for *Haemonchus contortus* [28] with females metabolizing benzimidazole anthelmintics more extensively than males and resistance of *H*. *contortus* females showing greater sensitivity to ivermectin than males [29] but gender-linked effects are not found routinely [30]. We advocate that gender, as a variable for parasitic nematodes should be investigated as early as possible because of potential therapeutic consequences.

### Conclusion

Our observations show that emodepside has potent, gender-dependent, inhibitory effects on the motility of *B*. *malayi* that is mediated by activation of SLO-1 potassium channels in adult *Brugia malayi* filaria. Importantly, splice variants and mutations in the RCK regions of the binding pocket for emodepside can alter the potency of emodepside. As the potency of an anthelmintic drug can be different against one life-cycle stage than another, in this particular case female less than that on the male macrofilariae, it is of major importance to define the dose-regimen based all stages that need to be eliminated with the treatment.

## Methods

### Parasite maintenance

*B*. *malayi* adult worms were obtained from the NIH/NIAID Filariasis Research Reagent Resource Center (FR3; College of Veterinary Medicine, University of Georgia, Athens, GA, USA). Adult worms were maintained in non-phenol red Roswell Park Memorial Institute (RPMI) 1640 media (Life Technologies, USA) supplemented with 10% heat-inactivated fetal bovine serum (FBS, Fisher Scientific, USA) and 1% penicillin-streptomycin (Life Technologies, USA). The worms were stored individually in 24 well microtiter plates containing 2 mL RPMI -1640 media containing L-glutamine and placed in an incubator at 37°C supplemented with 5% CO_2_.

### *Brugia malayi m*otility analysis

Movement of worms was analyzed in a 24-well microtiter plate using the Worminator system as explained by [31]. The output of the Worminator, Mean Movement Units (MMU), measures the pixel displacement of each worm over time and decreases to zero for stationary worms. Each worm was placed in a single well of the microtiter plate containing 1 mL of RPMI media containing L-glutamine. For emodepside concentration-response analysis, once the drugs were added the motility of the worms was recorded 0, 10, 20, 30, 40, 60, 90 and 120 minutes post treatment. Emodepside was dissolved in DMSO (final concentration of 0.1% (v/v)) and the same concentration of DMSO was used in control worms. For dsRNA experiments, either 30 μg/mL of *Bma-slo-1*, LacZ dsRNA or DNA/RNase free water were used. The movement of worms was recorded at 0, 24, 48, 72 and 96 hours post dsRNA treatment using the WormAssay v1.4 software. Motility of control worms was also recorded before the application of dsRNA. The worms were treated with emodepside after 96 hours of RNAi. The motility of the worms was recorded at 0, 15, 30, 45, 60, 90 and 120 minutes post treatment. % motility was calculated as a percentage ratio of motility of worms after treatment at each time point over motility of naïve worms.

### Dissection

Once all the worms were dissected, the recordings were performed at room temperature. The muscle cells and the hypodermis were exposed upon dissection by modifying the methods used for *C*. *elegans* [32, 33]. Sections of about 5mm were cut from the anterior region of the worm and placed in the recording chamber with bath solution (23 mM NaCl, 110 mM Sodium acetate, 5 mM KCl, 6 mM CaCl_2_, 4 mM MgCl_2_, 5 mM HEPES, 10 mM d-glucose, and 11 mM sucrose, pH adjusted to 7.2 with NaOH, ~320 mosmol). The base of the chamber was a 24 × 50 mm cover slip coated with a thin layer of Sylgard. The worm section was then glued along one side using Glushield cyanoacrylate glue (Glustitch Inc, Canada) thereby immobilizing it and then cut open longitudinally using a tungsten needle. The resulting ‘muscle flap’ was glued along the cut edge and the reproductive and the gut tissue were removed using fine forceps. The dissection was viewed under DIC optics (400X) using an inverted light microscope (TE2000U, Nikon, USA).

### Whole cell recording

Muscle flaps were incubated in 1 mg/ml collagenase (Type 1A) in bath solution for 15-120s and washed 10 times prior to recording. The patch-clamp technique was used to record whole-cell currents from the muscle flaps as explained in [34]. Patch pipettes were pulled from capillary glass (G85150T; Warner Instruments Inc., Hamden, CT, USA), fire polished and then filled with pipette solution (120 mM KCl, 20 mM KOH, 4 mM MgCl_2_, 5 mM TRIS, 0.25 mM CaCl_2_, 4 mM NaATP, 5 mM EGTA and 36 mM sucrose (pH 7.2 with KOH), ~315–330 mosmol). Pipettes with resistances of 3–5 MΩ were used. A 1 cm region near the tip of the electrode was covered with Sylgard to reduce background noise and improve frequency responses. Giga ohm seal was formed before breaking the membrane with suction. The preparation was continuously perfused in bath solution at 2 ml/min. The current signal was amplified by an Axopatch 200B amplifier (Molecular Devices, CA, USA) filtered at 2 kHz (three-pole Bessel filter), and sampled at 25 kHz, digitized with a Digidata 1440 (Molecular Devices, CA, USA).

### RNA extraction and cDNA synthesis

Worms were snap frozen and crushed into fine powder in a 1.5 mL micro-centrifuge tube using Kimble Kontes Pellet Pestle (Fisher Scientific, USA). Total RNA was extracted using TRIzol Reagent (Life Technologies, USA) according to the manufacturer’s instructions. About 1μg of total RNA was used to synthesize cDNA using SuperScript VILO Master Mix (Life Technologies, USA). Samples were either used to amplify DNA using PCR or stored at -20°C for later use.

### Synthesis and delivery of dsRNA

dsRNA was synthesized as explained in [20, 35]. Target and non-target T7 promoter labelled primers were amplified using the primers SSK 34F, SSK 34R, SSK 34Ft7 and SSK 34Rt7 for the target *Bma*-*slo-1*, LacZF, LacZR, LacZFt7 and LacZRt7 for the non-target LacZ. The sequences of these primers are shown in Table 2. Amplification was done using Techne PRIMEG (Bibby Scientific Limited, UK) with cycling conditions -95°C x 5 min, 35 x (95°C x 30s, 55°C x 30s, 72°C x 1 min), 72°C x 10min from sequence verified cDNA templates. dsRNA was synthesized T7 RiboMAX Express RNAi kit (Promega, USA) according to the manufacturer’s instructions. Concentration and purity of dsRNA were assessed using a spectrophotometer. Adult *B*. *malayi* were soaked in 30 μg/mL of dsRNA for 4 days. Worms were maintained in RPMI media as explained before. A part of the worm was cut for electrophysiology recordings and the rest were snap frozen in liquid nitrogen and stored at -80°C for transcript analysis.

**Table 2:** List of primers used. Shows a list of primers used in the study along with the description and nucleotide sequences.

Primer Name	Description	Sequence 5' - 3'
**SSK 34F**	Bma slo-1 dsRNA PCR 5'	GAAAACAGTGGTGATCCCTTC
**SSK 34R**	Bma slo-1 dsRNA PCR 3'	CCAATTAAGTCAGCTATTTCCGG
**SSK 34Ft7**	Bma slo-1 dsRNA PCR with t7 promoter 5'	TAATACGACTCACTATAGGGGAAAACAGTGGTGATCCCTTC
**SSK34Rt7**	Bma slo-1 dsRNA PCR with t7 promoter 3'	TAATACGACTCACTATAGGGCCAATTAAGTCAGCTATTTCCGG
**SSK 48F**	Infusion Bma slo-1 BamHI 5'	TACCGAGCTCGGATCCATGAGCGATGTATACCATCCTGG
**SSK 48R**	Infusion Bma slo-1 BamHI 3'	CTGGACTAGTGGATCCTCATAAGAAGTTTTTCCTTGGTTCG
**SSK 5F**	Bma GAPDH 5' Fwd	GACGCTTCAAGGGAAGTGTTTCTG
**SSK 5R**	Bma GAPDH 3' Rev	GTTTTGGCCAGCACCACGAC
**LacZF**	LacZ dsRNA 5'	CGTAATCATGGTCATAGCTGTTTC
**LacZR**	LacZR dsRNA 3'	CTTTTGCTGGCCTTTTGCTC
**LacZFt7**	LacZ dsRNA with t7 promoter 5'	TAATACGACTCACTATAGGGCGTAATCATGGTCATAGCTGTTTC
**LacZRt7**	LacZR dsRNA with t7 promoter 3'	TAATACGACTCACTATAGGGCTTTTGCTGGCCTTTTGCTC
**Bslo-1F1**	Bma Slo-1 isoform test 5’ 1	ATCCCTTCATGGGATTGGCG
**Bslo-1F2**	Bma Slo-1 isoform test 5' 2	CTCCGGACTGGTTAAATTTGTACC
**Bslo-1R**	Bma Slo-1 isoform test 3'	CTTGCCGGACACCAGTGGAAC

### Analysis of transcript levels

cDNA from dsRNA treated worms were amplified using target (*Bma slo-1*) and reference gene (*Bma gapdh*) primers–SSK 34F, SSK 34R, SSK 5F and SSK 5R (Table 2). These genes were amplified in triplicate by quantitative real-time PCR (qPCR) using the CFX96 Touch Real-Time PCR Detection System and SsoAdvanced Universal SYBR Green Supermix (Bio-Rad, USA). Cycling conditions used: 95°C x 10min, 40 x (95°C x 10s, 55°C x 30s). PCR efficiencies were calculated using the CFX96 Software Suite (Bio-Rad, USA). Relative quantification of target gene knock down was estimated by the ΔΔCt method [36].

### Cloning and expression in *Xenopus laevis* oocytes

Defolliculated *Xenopus laevis* oocytes were purchased from Ecocyte Bioscience (Austin, TX, USA). Full length *slo-1* was amplified using Platinum SuperFi Polymerase Master Mix (ThermoFisher, USA) in a thermocycler. Primers (SSK 48F and SSK 48R) were made with sequences flanking the expression vector pTB207 including the restriction site (BamHI) between which *slo-1* was inserted (Table 1). The amplicon was then purified using NucleoSpin Gel and PCR Clean-up kit (Macherey-Nagel) and cloned into pTB207 by using Infusion HD Cloning Kit (Takara Bio USA, Inc.) using the manufacturer’s guidelines. Upon cloning, the plasmids were sequence verified, linearized by MscI and purified for *in vitro* transcription using the mMessage mMachine T7 Transcription Kit (Ambion, USA). The cRNA was precipitated with lithium chloride, re-suspended in nuclease free water, and stored at -80°C. The cRNA was then injected into *Xenopus laevis* oocytes kept at 20°C for ~3 hours prior to injections in incubation solution (100 mM NaCl, 2 mM KCl, 1.8 mM CaCl_2_.2H_2_0, 1 mM MgCl_2_.6H_2_0, 5 mM HEPES, 2.5 mM Na pyruvate, 100 U/mL penicillin and 100 μg/mL streptomycin, pH 7.5). 15ng of *slo-1* cRNA was injected into the animal pole of the oocytes using a nanoject II microinjector (Drummond Scientific, PA, USA). The injected oocytes were transferred into 96-well culture plates containing 200μL incubation solution per well; each well contained one oocyte. Oocytes were incubated at 20°C for 5–6 days to allow for receptor expression, incubation solution was changed daily. Oocytes with membrane potentials less than -15 mV were excluded from recording. Oocyte recordings that failed (shown by a change in the holding current following wash) before the complete series of drug applications were excluded from the analysis.

### Two-electrode voltage-clamp

Two-electrode voltage-clamp electrophysiology was used to record currents produced by activation of the expressed Bma-SLO-1 channels. Recordings from water injected oocytes served as control experiments. Recordings were made using an Axoclamp 2B amplifier (Warner Instruments, USA) with the oocytes voltage-clamped at +20 mV, and data acquired on a computer with Clampex 10.3 (Molecular Devices, CA, USA). The microelectrodes used to impale the oocytes were pulled using a Flaming/Brown horizontal electrode puller (Model P-97, Sutter Instruments, USA) set to pull micropipettes that when filled with 3 M KCl had a resistance of 20–30 MΩ. The micropipettes tips were carefully broken with a piece of tissue paper in order to achieve a resistance of 2–5 MΩ in recording solution (100 mM NaCl, 2.5 mM KCl, 1 mM CaCl_2_.2H_2_O and 5 mM HEPES, pH 7.3). The low resistance pipettes allowed large currents to be passed to maintain adequate voltage-clamp.

Emodepside used in this study was obtained from Bayer Animal Health. Potassium channel inhibitor iberiotoxin from Sigma Aldrich (St. Louis, MO, USA). The drugs were solubilized in DMSO and diluted in recording solution.

### *In-silico* analysis and molecular docking

The molecular structure of emodepside was obtained from DrugBank (DB). Docking studies were performed using AutoDock (The Scripps Research Institute) as explained in [37]. AutoDock does not consider single bonds in non-aromatic cycles as rotatable bonds. To add more flexibility to the structure of emodepside, we used Marvin Sketch and the minimization under MMFF94 force field provided in the Marvin Suite (www.chemaxon.com/products/marvin/marvinsketch/). The ten least energy conformers were evaluated using pairwise root mean square deviations (RMSD) for atomic positions using PyMOL. Conformers 1, 2 and 4 were used for further docking analysis.

Molecular docking experiments were performed using AutoDock 4.2.6. Structures of filarial SLO-1 were modelled using Swiss Model (https://swissmodel.expasy.org). The structures were kept rigid and only the cytosolic region of monomeric SLO-1 was used for docking. Due to the absence of literature about the exact location of the binding site, the entire cytosolic region was taken into account for the computation. The grid box drawn using AutoGrid 4 included 126, 126 and 126 points in the x, y and z directions with a grid spacing of 0.586Å. For each ligand conformer, 25 independent computations were performed using Lamarckian genetic algorithm. All other parameters were set to default value. The pose with the least binding energy for each filarial species was compared and the interacting amino acids were mapped in AutoDock.

### Data analysis

Whole-cell patch clamp data were analyzed with Clampfit 9.2 (Molecular Devices, CA, USA) and GraphPad Prism 5.0 software. The peak current responses from whole-cell recordings were used for analysis. For two-electrode voltage clamp experiments, the response to 30 μM emodepside was used to normalize the concentration–response relationship. For whole worm concentration–response relationships, motility/minute was plotted against log concentration. Emodepside concentrations were log_10_ transformed before analysis. The log agonist vs. response equation (variable slope) was used to generate concentration response curves to calculate EC_50_ values. The responses were plotted as the mean ± Standard Error of Mean (SEM). Statistical analyses were performed on groups of values by using ANOVA to determine whether the group means were dissimilar; Bonferroni post-hoc tests were used for multiple comparison tests to determine whether there were significant differences between groups. Student’s *t*-tests were used for comparing a simple control with a test study effect.

## Supporting information

S1 Fig Effect of voltage-steps on emodepside activated currents in *B*. *malayi* muscle cells. **A:** A representative trace of voltage-activated outward-currents in naïve (**black**: top) and emodepside treated muscle preparations (**red**: middle), bottom trace shows the voltage-step protocol (10 mV steps: **green**), holding potential -40mV. The preparation was perfused with 1μM emodepside for 30 seconds prior to and during the voltage steps. **B:** Demonstrates the IV plot of control vs emodepside the treated preparation shown in **A**. **C:** Shows the activation curve for emodepside mediated mean ±SEM increase in conductance of the potassium channel currents for 5 experiments on 5 preparations like those shown in **A** and **B**. *G*_*max (Emodepside*)_ = 23 ± 1pS, *G*_*max (Control)*_ = 14 ± 2pS, *V*_*half (Emodepside)*_ = -2 ± 1 mV, *V*_*half (Control)*_ = 8 ± 1 mV, n = 5. There was little change in the slope factor. Note that *G*_*max*_ was increased by emodepside showing that the number of SLO-1 channels opening has increased and/or the maximum probability of them being open has increased; the voltage-sensitivity of the channel showed only a modest hyperpolarizing shift.
(TIF)

S2 Fig Emodepside currents blocked by SLO-1 K channel antagonist, iberiotoxin. **A:** Representative trace showing the inhibition (reversible on washing) of the emodepside induced current by 100nM iberiotoxin (IbTx). IbTx had no effect on its own. **B**: Bar chart showing mean ±SEM outward currents in presence of 300 nM emodepside and 300 nM emodepside with 100nM IbTx. IbTX significantly inhibits the outward currents induced by emodepside (p<0.005, paired Student’s *t-*test, n = 7).
(TIF)

S3 Fig Expression of *slo-1* isoforms in *Brugia malayi*. **A:** A diagram of the predicted isoforms (splice variants: a, b, c, e & f) of *slo-1* in *B*. *malayi*. Exons 2 and 14 are marked with a vertical arrow, ↓, **B:** Agarose gel showing expression of *slo-1* splice variants in single muscle cells of both male and female. AF (a & f): amplified using 5’ SL2 and a 3’ primer BsloR1; E (e): amplified using 5’ SL2 and 3’ primer BsloR2. **C:** Full-length *slo-1* cDNA from male and female worms cleaved by NcoI reveals the expression of *slo-1a* and *slo-1f* in female worms and *slo-1f* alone in male worms.
(TIF)

S4 Fig Selective knock-down of *slo-1a* transcript in female adult *B*. *malayi*. **A:** Transcript knock-down in female worms specific to *slo-1a*. Significant knock-down (86.72%) of *slo-1a* transcript was achieved in female worms while non-specific (*lacZ*) knock-down of *slo-1f* was 12.06% (p<0.01, Student’s *t-*test). n = 5 for each estimation using two biological replicate experiments. **B:** Shows no *slo-1f* transcript knock-down in adult male treated with *slo-1a* specific dsRNA. Male worms lack *slo-1a* and non-specific knock-down of *slo-1f* is similar to *lacZ* dsRNA treated control worms.
(TIF)

S5 Fig Expression of *slo-1* splice variants in female *O*. *volvulus*. **A:** A diagram of the predicted isoforms (splice variants) of *slo-1* in *O*. *volvulus* and the locations of the primers that were used to amplify the expressed isoforms. **B:** Table showing the different product sizes for the predicted isoforms when amplified using different the primer combinations. **C:** Agarose gel showing the expression of *slo-1* isoforms in cDNA synthesized from whole worm lysates in female *O*. *volvulus*. Amplicons were obtained at 537, 492 and 432bp indicating the expression of *slo-1a* and *slo-1d* splice variants.
(TIF)

S6 Fig Emodepside binding loops on the cytoplasmic domains of SLO-1. Cartoon showing *in silico* homology modelling of emodepside bound to the RCK regions of other filarial nematodes: **A:** *O*. *volvulus* (*Ovo* SLO-1A). **B:** *L*. *sigmodontis* (*Lsi* SLO-1) and **C:** *D*. *immitis* (*Dim* SLO-1).
(TIF)

S7 Fig Diagram of a single subunit of SLO-1 illustrating the location of the binding pocket location of emodepside. The SLO-1K channel is composed of a tetramer of subunits each of which have seven transmembrane regions (S0-S6), a pore forming region (P) and a cytoplasmic domain composed of an RCK1 region and an RCK2 region. Only one subunit is displayed. Both the RCK1 and RCK2 regions have calcium-binding sites (★ and ★); RCK1 also has a magnesium-binding region near the emodepside binding site.
(TIF)
