# Supplementary figures and images for: Emodepside has sex-dependent immobilizing effects on adult Brugia malayi due to a differentially spliced binding pocket in the RCK1 region of the SLO-1 K channel
Source: PLoS Pathog. 2019 Sep 25;15(9):e1008041. doi: 10.1371/journal.ppat.1008041 (PMC6779273; doi:10.1371/journal.ppat.1008041)

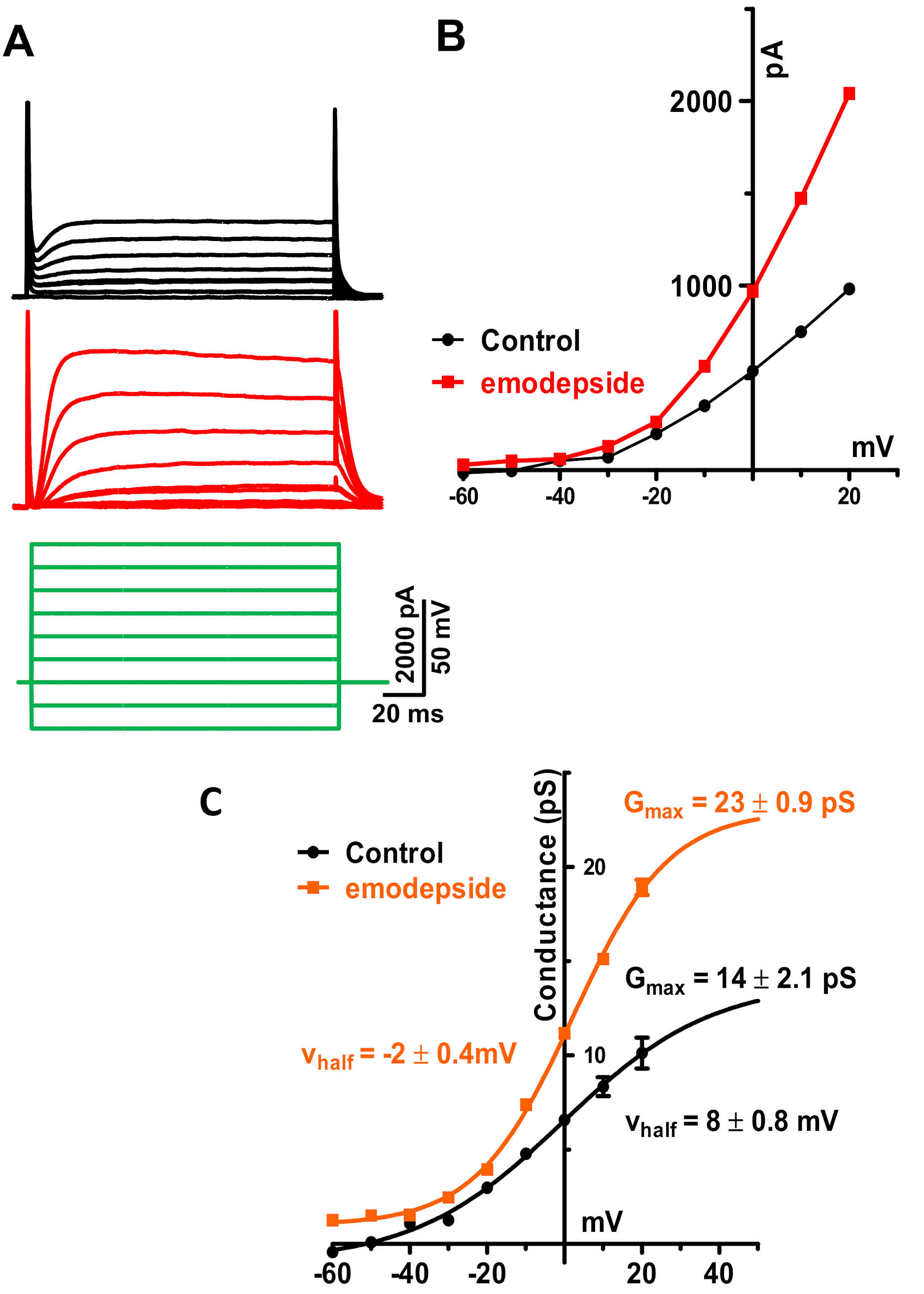

Supplement: S1 Fig — A: A representative trace of voltage-activated outward-currents in naïve (black: top) and emodepside treated muscle preparations (red: middle), bottom trace shows the voltage-step protocol (10 mV steps: green), holding potential -40mV. The preparation was perfused with 1μM emodepside for 30 seconds prior to and during the voltage steps. B: Demonstrates the IV plot of control vs emodepside the treated preparation shown in A. C: Shows the activation curve for emodepside mediated mean ±SEM increase in conductance of the potassium channel currents for 5 experiments on 5 preparations like those shown in A and B. Gmax (Emodepside) = 23 ± 1pS, Gmax (Control) = 14 ± 2pS, Vhalf (Emodepside) = -2 ± 1 mV, Vhalf (Control) = 8 ± 1 mV, n = 5. There was little change in the slope factor. Note that Gmax was increased by emodepside showing that the number of SLO-1 channels opening has increased and/or the maximum probability of them being open has increased; the voltage-sensitivity of the channel showed only a modest hyperpolarizing shift. (TIF) [file ppat.1008041.s001.tif]

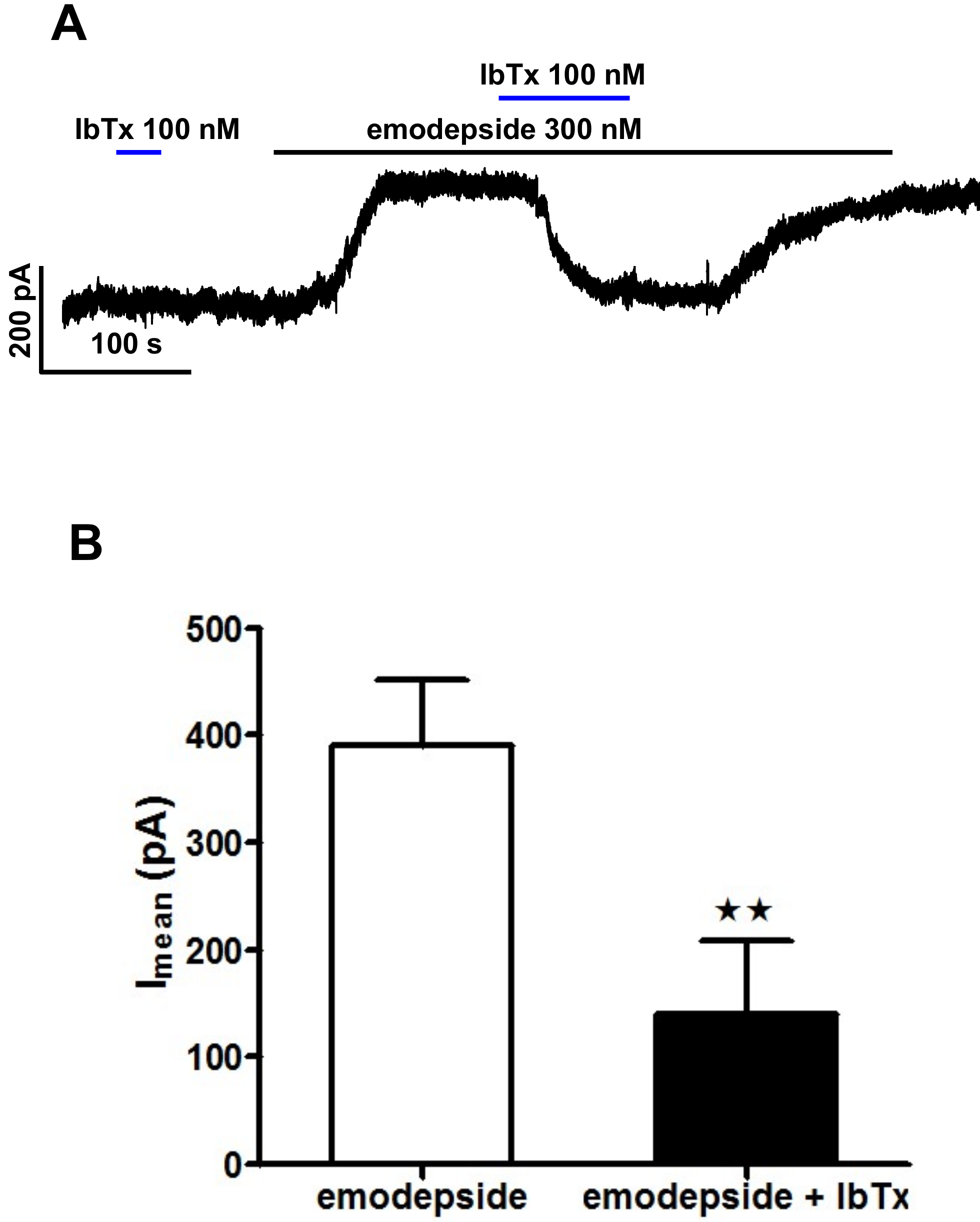

Supplement: S2 Fig — A: Representative trace showing the inhibition (reversible on washing) of the emodepside induced current by 100nM iberiotoxin (IbTx). IbTx had no effect on its own. B: Bar chart showing mean ±SEM outward currents in presence of 300 nM emodepside and 300 nM emodepside with 100nM IbTx. IbTX significantly inhibits the outward currents induced by emodepside (p<0.005, paired Student’s t-test, n = 7). (TIF) [file ppat.1008041.s002.tif]

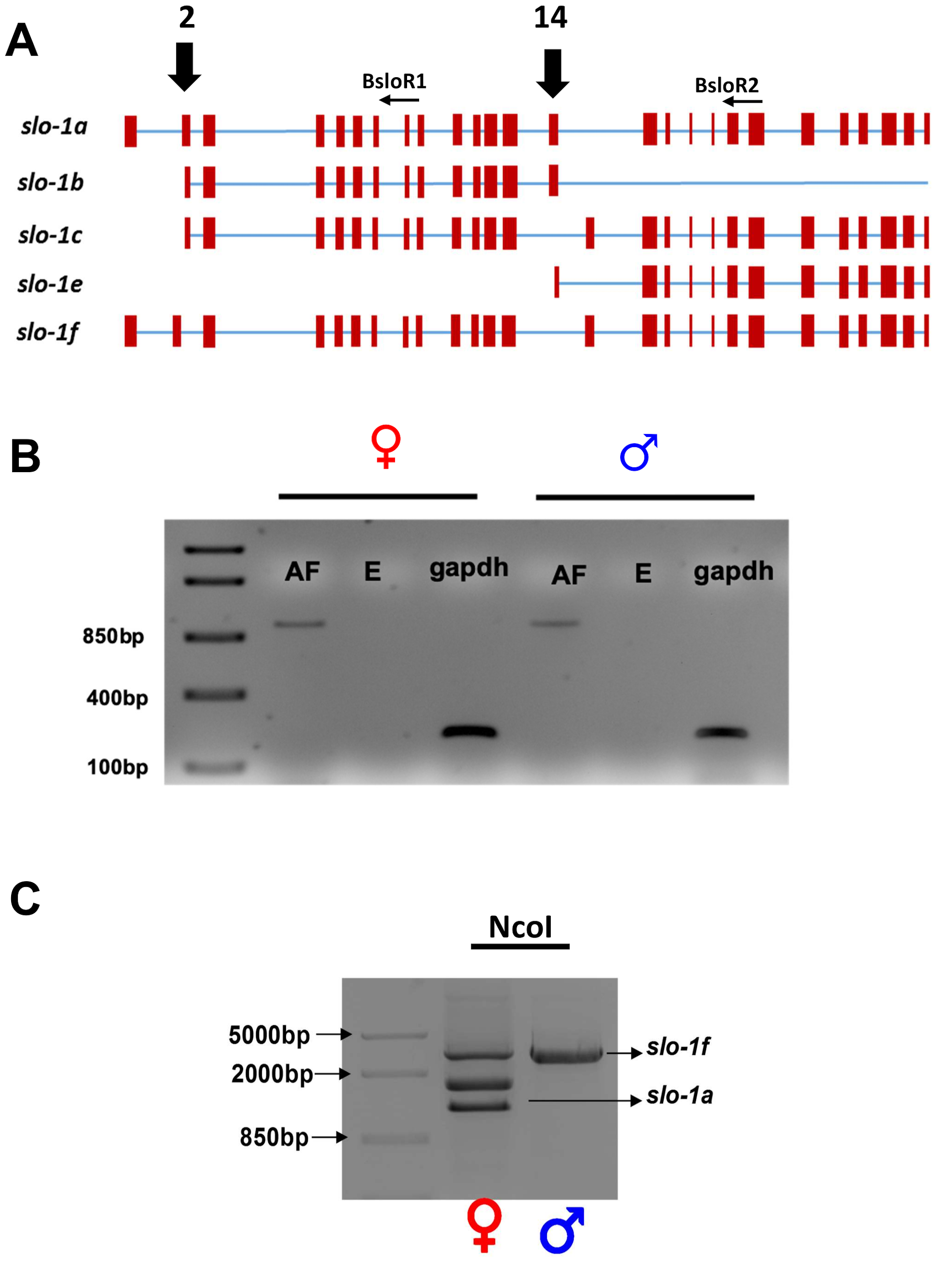

Supplement: S3 Fig — A: A diagram of the predicted isoforms (splice variants: a, b, c, e & f) of slo-1 in B. malayi. Exons 2 and 14 are marked with a vertical arrow, ↓, B: Agarose gel showing expression of slo-1 splice variants in single muscle cells of both male and female. AF (a & f): amplified using 5’ SL2 and a 3’ primer BsloR1; E (e): amplified using 5’ SL2 and 3’ primer BsloR2. C: Full-length slo-1 cDNA from male and female worms cleaved by NcoI reveals the expression of slo-1a and slo-1f in female worms and slo-1f alone in male worms. (TIF) [file ppat.1008041.s003.tif]

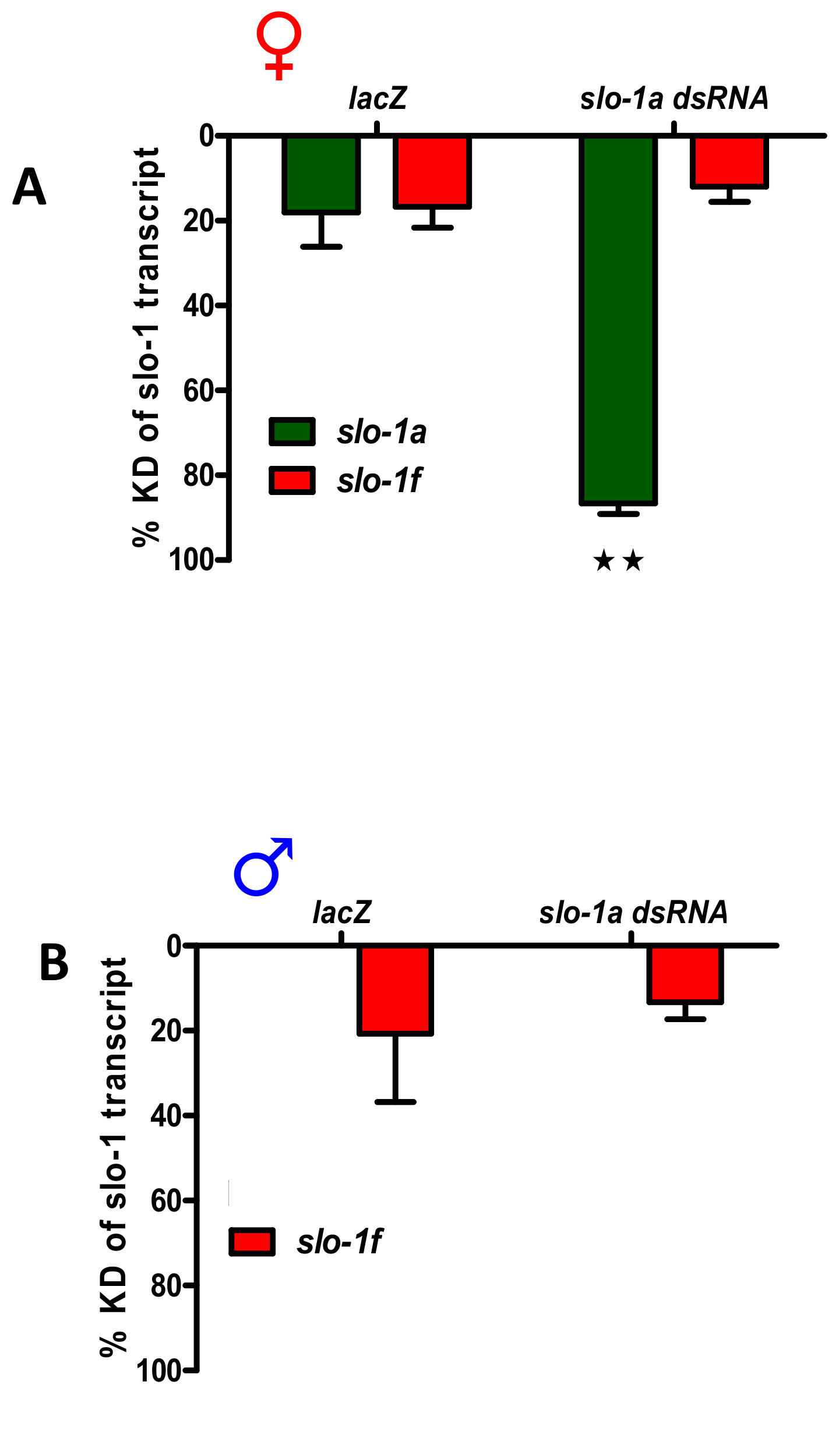

Supplement: S4 Fig — A: Transcript knock-down in female worms specific to slo-1a. Significant knock-down (86.72%) of slo-1a transcript was achieved in female worms while non-specific (lacZ) knock-down of slo-1f was 12.06% (p<0.01, Student’s t-test). n = 5 for each estimation using two biological replicate experiments. B: Shows no slo-1f transcript knock-down in adult male treated with slo-1a specific dsRNA. Male worms lack slo-1a and non-specific knock-down of slo-1f is similar to lacZ dsRNA treated control worms. (TIF) [file ppat.1008041.s004.tif]

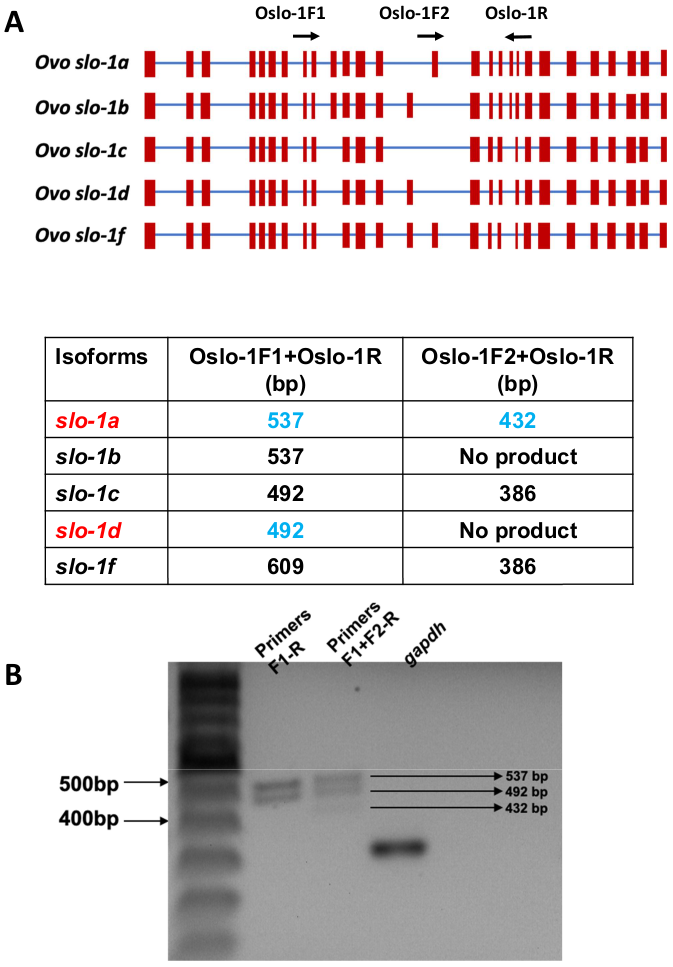

Supplement: S5 Fig — A: A diagram of the predicted isoforms (splice variants) of slo-1 in O. volvulus and the locations of the primers that were used to amplify the expressed isoforms. B: Table showing the different product sizes for the predicted isoforms when amplified using different the primer combinations. C: Agarose gel showing the expression of slo-1 isoforms in cDNA synthesized from whole worm lysates in female O. volvulus. Amplicons were obtained at 537, 492 and 432bp indicating the expression of slo-1a and slo-1d splice variants. (TIF) [file ppat.1008041.s005.tif]

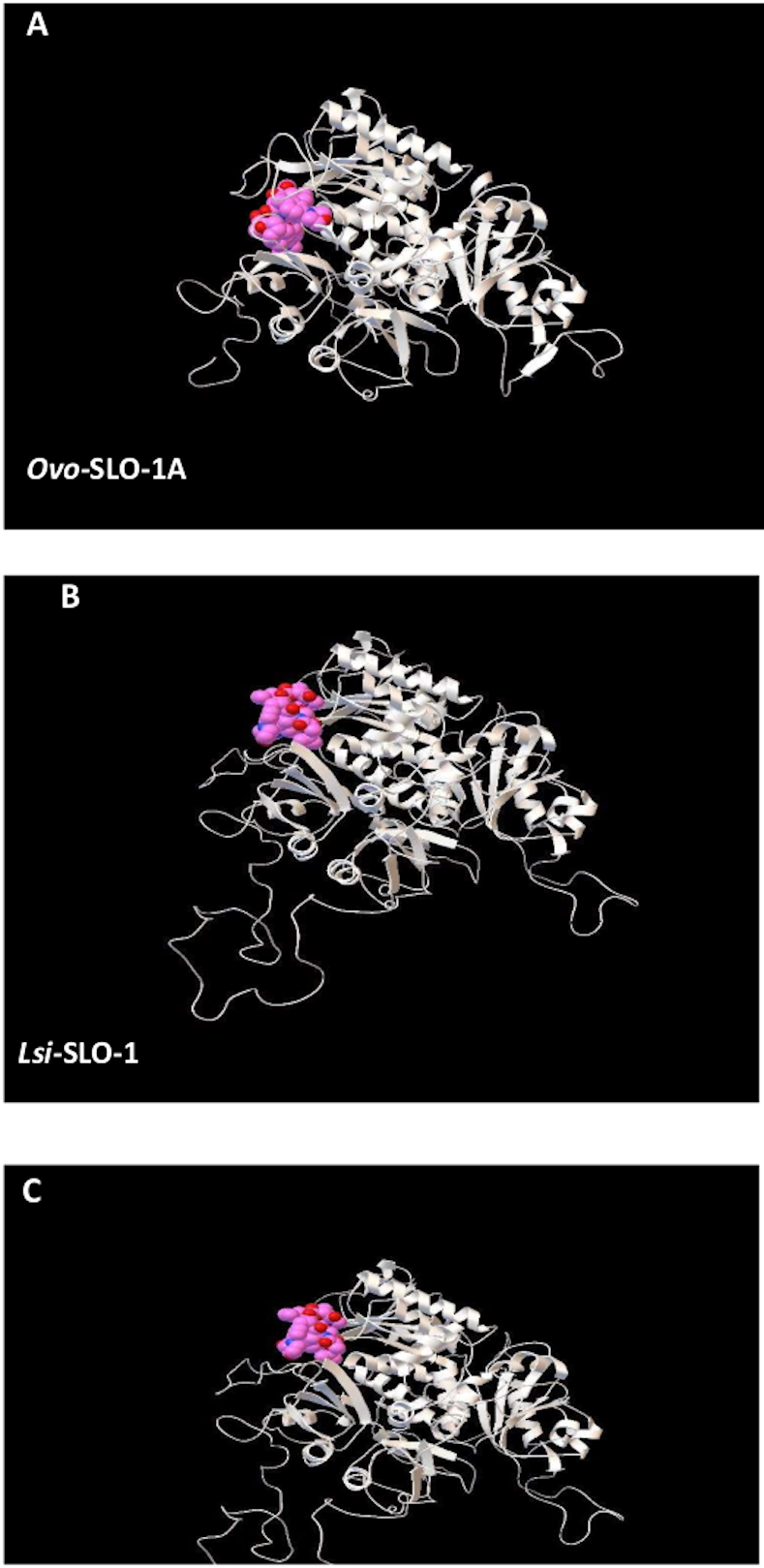

Supplement: S6 Fig — Cartoon showing in silico homology modelling of emodepside bound to the RCK regions of other filarial nematodes: A: O. volvulus (Ovo SLO-1A). B: L. sigmodontis (Lsi SLO-1) and C: D. immitis (Dim SLO-1). (TIF) [file ppat.1008041.s006.tif]

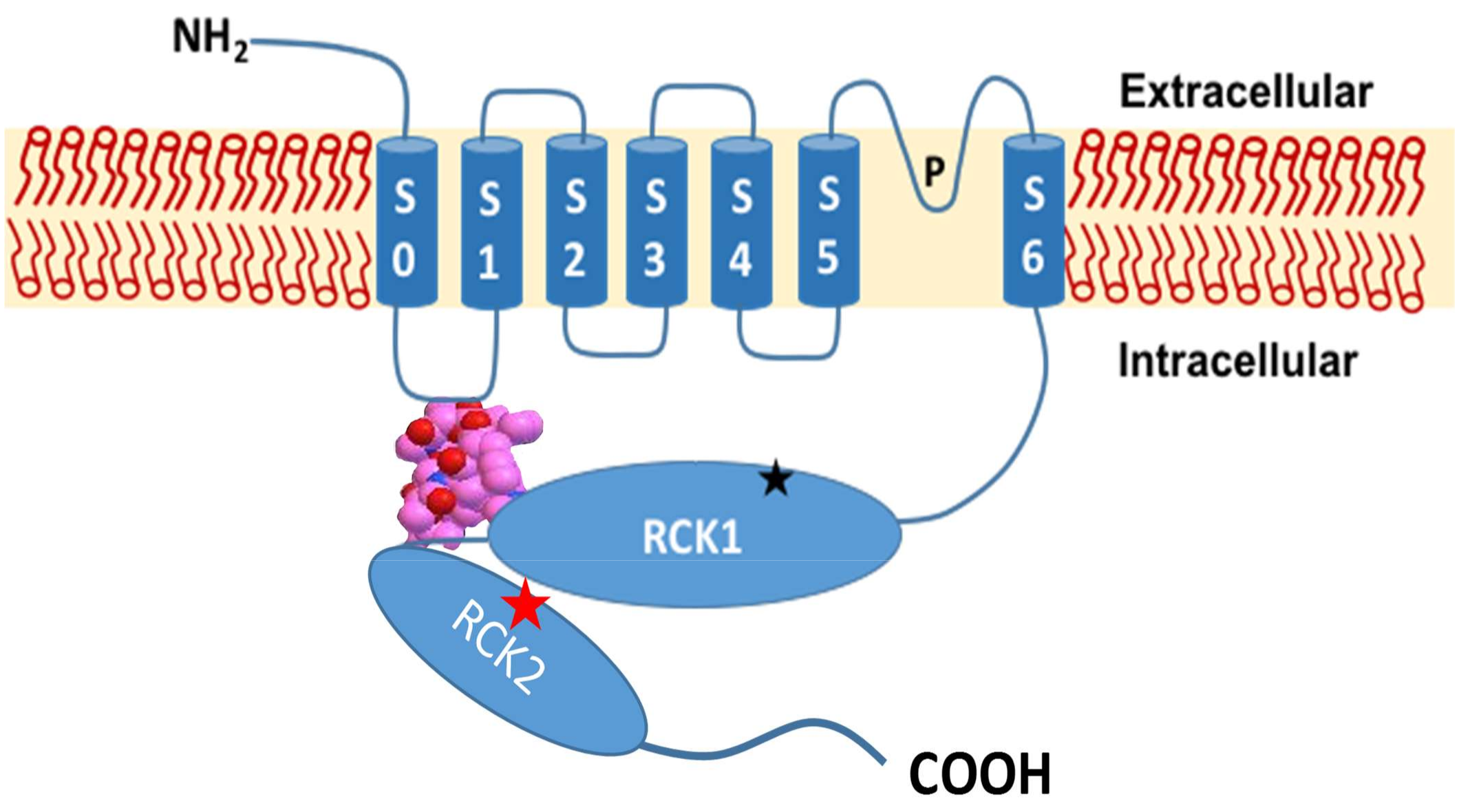

Supplement: S7 Fig — The SLO-1K channel is composed of a tetramer of subunits each of which have seven transmembrane regions (S0-S6), a pore forming region (P) and a cytoplasmic domain composed of an RCK1 region and an RCK2 region. Only one subunit is displayed. Both the RCK1 and RCK2 regions have calcium-binding sites (★ and ★); RCK1 also has a magnesium-binding region near the emodepside binding site. (TIF) [file ppat.1008041.s007.tif]
